# Self and identity in women with symptoms of borderline personality: A qualitative study

**DOI:** 10.3402/qhw.v11.30490

**Published:** 2016-03-23

**Authors:** Gillian Agnew, Ciarán Shannon, Tina Ryan, Lesley Storey, Catherine McDonnell

**Affiliations:** 1 School of Psychology, Queen's University Belfast, Belfast, UK; 2 Southern Health and Social Care Trust, Northern Ireland, UK; 3 Western Health and Social Care Trust, Northern Ireland, UK

**Keywords:** Personality disorder, identity, self, lightly structured interviews, qualitative analysis, thematic analysis

## Abstract

Identity disturbance has been suggested to be a core feature of borderline personality disorder (BPD). However, there is little known about the identity of individuals with symptoms of BPD from the participant's perspective. This study availed of in-depth lightly structured life story interviews with five female participants. Thematic analysis was utilized to derive three themes of identity: connection, distance between us, and hurt and healing. Results provided support for multiple and flexible conceptualizations of identity in comparison to the idea of a unitary self/identity. Results also suggested that participants were able to establish differing connections to others ranging from disconnection to intimacy and care. Participants reported that their identities were impacted upon by historical and current family/relationship dysfunction, but life stories also illustrated the positive impact of healing relationship experiences. Findings provide support for psychological theories that consider a multiple and relational self/identity and the empowerment of healthy aspects of the self in BPD recovery. Studies that assess the association between insight and change may further our knowledge into this complex population.

The concepts of self and identity disturbance have been at the centre of most current theoretical frameworks used to understand borderline personality disorder (BPD). The “self” in common discourse is used to describe when something is “about me.” However, it is also a mental activity requiring that there is a self to consider thinking “about me.” Identity as a term is understood as a way of making sense of some part of the self (Oyserman, Elmore, & Smith, 2012). It has been characterized as the broadest category relating to the self and includes one's sense of self within the context of the socio-cultural environment, ideal self-representations, role relationships, and explicit/implicit worldviews (Westen & Cohen, 1993).

Self and identity are concepts used interchangeably within the literature, and they have been frequently undefined leading to difficulties in study comparison and differences in concept meaning between and within studies. Variances also exist in whether the terminology is referring to a singular or plural idea, for example, multiple selves and identities. The assumption of stability within the self and identity is contradicted by the flexibility, context sensitivity, and dynamic production of the self as a mental construct, for example, often a choice that is thought of as self-congruent in one situation is not in another (Oyserman et al., 2012).

BPD is categorized as a personality disorder (PD) consisting of nine diagnosable criteria (APA, 2013). Up to 6% of the general population has been estimated to meet criteria for BPD (Grant et al., 2008). The BPD literature largely describes individuals with BPD as suffering from identity disturbance, for example, chronic feeling of emptiness, dissociative states, and poorly integrated self-images (Paris, 2005). BPD is a highly controversial disorder and is commonly comorbid with other symptomology (Barone, Fossati, & Guiducci, 2011).

A small number of psychotherapies have been developed more recently offering specific methods for the treatment of BPD. These psychotherapies and their underlying theories largely hold the idea of multiple selves at the centre of their understanding of BPD difficulties (Bateman, Ryle, Fonagy, & Kerr, 2007; Linehan, 1987). For example, the conceptualization of a multifaceted self can be observed theoretically in mentalization theory (Bateman & Fonagy, 2010) and the ability to understand the mental states of others. This can be diminished at times leading to dissociation and concreteness of thought. The idea of a dialectical self within dialectical behaviour theory in the divisions between an emotional and rational self (Palmer, 2002) and also within schema therapy and the interaction between five borderline schema modes (Kellogg & Young, 2006). Finally, the multiple self-states model of BPD in cognitive analytical theory centralizes a structural dissociation between reciprocal roles (Ryle, 1997). In comparison to each other, these highly developed theories suggest that poor connections between parts of the self can explain the core and variable features of BPD. For example, features of BPD include frantic efforts to avoid real or imagined abandonment, affective instability due to a marked reactivity of mood, self-destructive behaviours, and a pattern of unstable and intense interpersonal relationships (Levy, 2005). Although commonalities exist between these differing theories, there are also some differences in their underlying assumptions, emphases, and links to contrasting therapeutic techniques (Bateman et al., 2007).

Qualitative research into the construct of the identity of young people and adults with BPD symptomology has grown in recent years with some research focusing on identity from the participants’ perspective (Chiesa, Drahorad, & Longo, 2000; Dammann, Hügli, & Selinger, 2011; Miller, 1994). One empirical study using clinical self-report found evidence for a multifaceted construct of identity including role absorption (self-definition based on a single role), painful incoherence, inconsistency (incoherence in thoughts, feelings, and behaviours), and a lack of commitment (jobs or values). This study was restricted in a way as it tested the assumptions of clinicians as opposed to how participants report identity disturbance directly (Wilkinson-Ryan & Westen, 2000). A qualitative study assessed individuals with BPD symptoms and their narrative identity using a narrative analysis. They found themes relating to disempowerment, a lack of connection fulfilment, and narrative inconsistency in participant stories (Adler, Chin, Kolisetty, & Oltmanns, 2012).

The studies discussed above have used a variety of qualitative assessments, sample sizes, age ranges, and assessment measures to derive findings (Adler et al., 2012; Chiesa, et al., 2000; Dammann et al., 2011; Miller, 1994; Wilkinson-Ryan & Westen, 2000). Therefore, comparability is problematic. Despite identity disturbance being described as a core symptom of BPD, the research studies in this area are limited, and the quality of research methodology is variable. The research that has been completed within this underresearched domain overall seems to suggest that difficulties in self and identity are largely present in BPD. Qualitative studies, in particular, seem to suggest that it is skewed towards negative attributions of the self and others. However, very little research into the understanding of identity from the participants’ perspective has been undertaken (Miller, 1994).

The aim of this study was to contribute to the gap between the existing research in exploring the concept of self/identity from the individual's perspective.

## Method

### Participants

Recruitment of participants took place via mental health professionals working within the multidisciplinary community mental health teams (CMHT), that is, community psychiatric nurses, social workers, and occupational therapists. CMHT work with adults aged 18–65 with severe and enduring mental health difficulties. To enable recruitment, a brief description and rationale of the study were presented to the CMHTs by the first author. Participants were then invited to take part in the study by CMHT members if they were over the age of 18, reached at least three criteria for BPD, were aware that they had a working BPD diagnosis, and were involved with mental health services. The exclusion criteria are as follows: intoxication or acutely suicidal at the time of the interview.

Staff acted as gatekeepers and discussed the study with service users on their caseload who they believed met inclusion/exclusion criteria for the study. After ethical consideration, a decision was made not to gather information from staff or participants who did not wish to participate in the study. Service users interested in the study were given an information sheet, consent form, and free post envelope by the CMHT member to consider independently and post if they wished at their convenience. This method was selected to minimize the impact of social desirability on informed consent, to provide service users with information, and allow a professional with specific information about a client to assess whether service users may meet inclusion/exclusion criteria.

### Measures

#### Screening interview

The population that participants were selected from was a general adult mental health service. A screening interview was included to confer suitability for inclusion in the study based on the nature and extent of each participants’ personality functioning. Participants attended a screening interview with a Consultant Psychiatrist, who was independent of the study and had no clinical involvement with participants. During the screening interview, the SIDP-IV was administered. Individuals also met three or more symptoms for other PDs. It has been suggested that a threshold of three criteria within the Structured Interview for DSM-IV Personality (SIDP-IV; Pfohl, Blum, & Zimmerman, 1997) for BPD represents a cut-off score with clinical importance (Clifton & Pilkonis, 2007; Oltmanns & Gleason, 2011). The SIDP-IV is a semi-structured interview that uses non-pejorative questions to examine behaviour and personality traits. It assesses 10 DSM-IV Axis II personality disorders. The SIDP-IV is organized by topic sections to allow a more natural conversation. Questions follow a yes or no format that is followed up by open-ended questions to explore answers rated on a 3-point scale.

In total, six participants returned consent forms with contact details and were contacted by the first author to arrange interviews. A participant completed the screening interview but was unable to attend the research interview, and this participant was omitted from the study. Table I illustrates information about the five female participants that were recruited. Pseudonyms were given to each participant to maximise confidentiality.

**Table I:** Demographic and personality information of participants.

Participant	Age	Status	Children	SIDP-IV (3+PD symptoms)
1	30–35	Cohabiting	0	BPD, PARAND, SZOID, STYPL, AVOID, DEPEN, DEPRS, SLFDF, NEGTV
2	40–45	Widowed	3	BPD, OBCMP, SLFDF, DEPRS
3	30–35	Cohabiting	2	BPD, PARAND, SZOID, STYPL, DEPEND, DEPRS
4	30–35	Cohabiting	1	BPD, AVOID, SLFDF, DEPRS
5	30–35	Single	1	BPD, DEPEND, NEGTV

BPD: borderline; PARAND: paranoid; SZOID: schizoid; STYPL: schizotypal; AVOID: avoidant; DEPEN: dependent; DEPRS: depressive; SLFDF: self-defeating; NEGTV: negativistic; OBCMP: obsessive compulsive.

### Demographic and personality information of participants

#### Researcher position

The first author is a 28-year-old white Northern Irish female clinical psychologist. From her clinical experience, working with people with a diagnosis of BPD, she questioned if a discrepancy existed between the theories she used to explain the behaviours associated with BPD (psychodynamic and cognitive behavioural) and the individuals who she came into clinical contact with.

The research adopted an objectivist epistemological position within this study, that is, there is a “real” objective, knowable truth. The methodology reflects this position through the minimization of bias or influence over outcome, for example, lightly structured interview format and removal of the third stage, use of researchers with no clinical involvement with participants, and acknowledgement and consideration of researcher position throughout data collection and analysis (credibility checks).

#### Research interview

Participants took part in a research interview consisting of a face-to-face, in-depth, lightly structured life story interview (Wengraf, 2001, p. 111–118) conducted by the first author who was blind to the results of the screening interview and had no clinical involvement with participants. The interview technique originating from Wengraf (2001) was modified and employed as it used a lightly structured interview format, that is, one narrative inducing question and a two-stage interview format. Initially, the interview technique comprised a three-stage interview format. However, for this study, the third phase was removed as the study focus was from the participant's perspective and, therefore, did not wish to guide the data based on theoretical expectations. This format was selected as it maximizes the assessment of self/identity by allowing participants to choose what is relevant to tell the researcher and emphasize the meaning of an individual's experience of his or her “system of relevance” (Hollway and Jefferson, 2000). The lengths of the interviews ranged from 60 to 120 min. Due to the time constraints if individuals had difficulty eliciting a narrative a prompt could be used. A participant availed of this.

### Data analysis

Interviews were audio-taped and transcribed verbatim by the first author. During this process initial thoughts and impressions were noted down. The data were listened to and read several times to improve transcription accuracy. This process also facilitated data immersion (Braun & Clarke, 2006). The first author thematically analysed transcripts of the interviews with credibility checks being carried out by the second and fourth authors throughout each stage of the thematic analysis specified by Braun and Clark (2006).

Credibility checks were conducted to ensure that the development of codes and themes at each phase were valid and connected to the research question and the data set. Within the transcripts, all of the participants were given pseudonyms and any identifiable information altered to maximize confidentiality. The concept of identity (Westen & Cohen, 1993) was focused upon during the analysis, and the results and discussion will consider both ideas about identity and the self.

The interview method (Wengraf, 2001) encourages the participant to contextualize their life experience in detail. The interview method enabled a latent thematic analysis to be conducted. This goes beyond the semantic content of the data and allows the identification and examination of the underlying ideas, assumptions, conceptualizations, and ideologies that are theorized as shaping or informing the semantic content of the data (Braun & Clark, 2006).

Within this study, thematic analysis was chosen over other forms of qualitative analysis such as grounded theory, narrative analysis, biographic narrative interview method and interpretative phenomenological analysis because they are underpinned by particular epistemological orientations. The thematic analysis applies to a wide variety of data sources and, in this case, the technique employed in the research interview, that is, lightly structured interview, allowed the author to use inductive and deductive approaches to themes, driven by the identified research questions.

### Ethical approval

Ethical approval for the study was granted by a local research ethics committee regulated by a statutory research governance framework (ORECNI reference 13/NI/0030 24/04/13). Participants received an information sheet informing them of the rationale for the study and what participation would involve. Voluntary participation, rights to withdraw from the study, confidentiality and limits, and potential risks were also explained. Written informed consent was obtained from each participant.

## Results

Overall from the analysis, three major themes were generated, and each theme contained subordinate themes within them. Results will provide a brief description of some key subordinate themes. The presentation of results has focused on each major theme using excerpts from participant interviews to demonstrate theme meaning. Deeper analysis of the thematic relationships is focused upon within the discussion section when considering contemporary self/identity theories.

### Connecting to myself

#### Subordinate themes

Participants described themselves in multiple connecting and disconnecting ways.

*Connecting*. Some participants referred to their ability to hold on to connection to their sense of self including their early life experiences, memories, behaviour during recent events, and the coherence of self across situations.

*Conflicting*. Participants also described an internal struggle within themselves involving ideas of morality and wickedness, goodness and badness, and childishness and adultness.

*Cyclical*. This relates to patterns of self and behaviour. Participants described alternating moods and swinging from periods of inactivity, seclusion, and negativity to periods of complete positivity, activity, and involvement with others.

*Disconnected*. Participants spoke about the blocking, disconnection, and a glazing over of some traumatic events in their lives and disconnection between aspects of childhood and adulthood. Some participants described thinking about some negative life experiences as if it happened to someone else.

#### Major theme

Participants spoke about how they viewed and understood themselves. Participants demonstrated the ability to connect to their past experiences and relationships as children with their adult identity. Gina described how she experienced her father in childhood in a cyclical way and she seemed to connect this to her adult life in her excerpt relating to the subordinate theme cyclical identity.

When daddy was drinking, daddy was good fun, daddy was in a great mood, and the whole house just lifted. When daddy wasn't drinking my mum was in a really really vile mood. Then when daddy was sober, he was in a really bad mood, but my mum was in a great mood. So I think that your whole sort of mood shifted all the time and that it carried over into my adult life as well.

Within all participant narratives, there was an illustration that they felt lost, unreal, or conflicted. Susan's excerpt from the subordinate theme conflicting highlighted how at times she felt broken and destructive.

If somebody said come and sleep with me there is no like this is wrong, where are your morals. It's just all gone. I have another part of me that is really rigid and morals, and you don't do this. I don't get that. It's like a constant battle.

There was at times an extreme lack of connection in participant narratives. This is illustrated in an excerpt from the subordinate theme disconnected when Jane described a period of change within herself, her environment, and her family.

My mother took ill … diagnosed with cancer and within eleven days she died. So I came home and the night of the funeral I went straight back. I really had no time to think. I thought when I came home everything was going to be sorted. I was going to get on really well with my life but within a week, I was back in the psychiatric units and getting injections. I was just completely off the walls.

### Distance between us

#### Subordinate themes

The subordinate themes suggested that participants maintained differing proximities to others varying between confusion about self-separateness from others to intimacy.

*Self/other blurring*. This related to participants’ description of confusion and a difficulty separating themselves from others emotionally and the impact of others emotional state on their emotional state.

*Hiding from others*. Participants described how they at times wanted to keep the self hidden from others for fear of being judged negatively, being hurt, or abused.

*A different way*. Participants identified relationships with others that enabled connection, intimacy, and care. This subordinate theme also illustrated growth with others and improving and changing connections over time.

#### Major theme

Participants spoke about confusion and blurring of the physical, emotional, and psychological lines that separated themselves from others. Participants reported this in different ways but that this seemed to have an impact on their ability to regulate many aspects of their lives. Gina described an example of the subordinate theme self and other blurring when describing an interaction between her and her son.

My son, he had come home from work. I just felt this need to make him happy because I thought if I can't cheer him up and get him feeling up then I'm going to go down and within an hour of being in the same room with him being down I did go really down, and he bounced back, and he was fine … but my mood stayed.

All participants spoke about times when they attempted to hide the physical and psychological self from others. For Joan, this notion of hiding from others was to keep herself safe and communicate her fear to her abuser.

We used to have a cupboard under the stairs, and I used to hide in there because that was where the hoover and all was. I used to think if I hide in there he will not catch me he will not know … but he always knew.

Gina conversely conveyed an intense distress about her aloneness in the subordinate theme of barriers preventing connection when describing her inability to ask for help and an uncertainty about whether or not others could help.

I don't feel there is anybody at all that I can really turn to … that is probably my fault … I find it difficult to really talk to let things out fully … there are probably loads of people if I rung them up they would be there … but I can't get out to them.

Similarly, to the connecting to myself theme, a pattern of relating to others in a cyclical repeated fashion was identified. Jane described pushing away her relationships with romantic partners in her life as following a pattern shifting between depending heavily on them and pushing them away.

I thought I'm going to push her away now and see does she still love me, so I remember cutting my arms really badly from the wrist to the shoulder and saying to her look do you still love me now. Do you still love me now? And I got to hospital but testing her so see how far I could push her.

Participants described some occasions in which they demonstrated compassion, care, and intimacy towards others. Gina described a connection with others in her intimate work with young horses.

I work quite a lot with young horses that have never been handled. That is probably the one single time that I feel that I am there. I have a real affinity with them I don't know I can see something of myself in them. And every now and again I have to try and remember that feeling. I would love to bottle that feeling.

### Hurt and healing

#### Subordinate theme

They highlighted some ways participants’ experienced the world and relationships with others.

*Harsh and violating*. Participants described numerous relationships in life when they had been treated badly. Participants recounted lives interrupted with family and relationship dysfunction.

*Critical and controlling*. This subordinate theme depicted a pervasive sense of helplessness and rejection about others who were pervasively critical and controlling in her relationship with them.

*A different way*. Participants spoke about more recent changes in relationships which have provided a glimpse into different ways to relate to others and acceptance of more positive ways in which others can relate to them.

#### Major theme

All participants described negative and abusive relationships with others including child abuse, domestic abuse, rape, etc. They also spoke about critical and controlling relationships with parents and significant others. However, participants also reflected on times when they experienced helpful relationships and hopefulness for the future. Joan described severe and violating situations in which she was sexually abused by her father and brothers throughout her life.

When I was about eight mm my father started to mm sexually abuse me mm, and that was mostly on a daily basis. My brother [name] he was sixteen. I went into his bedroom to put away the socks. I was reaching over to put the socks in the top bunk and he eh reached out and grabbed me and started touching me down below. I thought right that is it. It will not happen again, and then it did.

Joan seems to relay a sense of the inevitability of abuse and an inability to prevent it. All participants described experiences of relationships within childhood and subsequently adulthood that was critical and controlling. Susan speaks about her mother as a controlling force in her life.

All the time. She had to tell you what to do all the time. You were criticised for every single thing. You might think that was an exaggeration, but it's not every time you opened your mouth mm you had done something wrong. I never spoke back to her.

Despite the difficult experiences participants also expressed differing levels of hopefulness for the future. Sarah at the end of her narrative described feeling balanced and hopeful for the future.

I'm a bit more balanced out now … because somewhere I want to do something yet with this life of mine you know … I do believe even for people with borderline personality there is a way forward if you let it.

Jane discussed a tentative changing relationship with her father, introducing them both to a different way of relating to each other.

My father. You know what he would do when I was living there is he would keep the heat on, he collects my prescription, and he gets me a bar of chocolate along with it. Just wee things he does that I know that he really cares.

## Discussion

All themes generated were continuum based. This highlighted the broad and diverse nature of how individuals experience symptoms associated with BPD. Comparisons of major themes emphasized the interacting and parallel nature of the themes with one another, for example, the self-relationship and its impact on how an individual relates to others and their life experiences. Specifically, the notions of different levels of connection, being engaged in a cyclical pattern but unable to change, of experiencing hurt caused by others, and of experiencing hopefulness are strong thematic comparisons identified throughout the analysis.

### Connecting to myself

This theme may provide support to theories that conceptualize a multiple and flexible self/identity due to the continuum-based themes generated. It challenges the idea of a unitary self and the concrete idea that an individual's identity/self may be intact or broken. Individuals with BPD may have differing levels of identity coherence and differing abilities at times to connect with themselves and others. This is highlighted in contemporary psychological BPD theories that have suggested BPD difficulties are related to differing levels of self-connection, for example, dissociation and self-states. For example, cognitive analytical therapy and the multiple self-states model of BPD identifies a structural dissociation between reciprocal roles (Ryle, 1997). This theory highlighted that the self is a multiple concept as individuals acquire a flexibility to mobilize a range of responses and choices depending on the context. “Normal” multiplicity may include the manifestation at different times of contradictory patterns but in general, there are stable links between patterns. However, in BPD exposure to adversity and predisposition result in a self that is fragmented and discontinuous with individuals demonstrating abrupt switches between states and potentially little recollection of them (Ryle, 2007).

The extreme multiplicity of the self in BPD seems to help explain the descriptions of the self in participants as disconnected. Results also highlight the most positive connections found within participants’ narratives. Theories have also conceptualized and integrated healthy adult modes to incorporate the part of individuals with BPD, who can have positive and connected relationships with the self and others, for example, Schema therapy and mentalization based therapy.

### Distance between us

Criteria assessing BPD recognizes difficulties making and maintaining stable relationships with others (APA, 2013). Conversely, the results of the current study identify a continuum of connections. Results have suggested that links to others have led to experiences of intimacy and care. These findings lend support to some contemporary theoretical perspectives that highlight the potential difficulties of individuals with BPD but also recognize the many opportunities and experiences of more positive relationships. For example, the mentalization model theorizes that experiences of early trauma can impact on an individual's ability reflect on the mental state of self and others. With individuals with BPD, Fonagy and colleagues propose that emotional arousal is related to the activation and deactivation of automatic and controlled mentalization functions. Therefore, theoretically there are times when an individual with BPD is not under emotional stress and can consider the possible thoughts and feelings of self/others. However, when emotionally aroused their reflective capacity can be restricted. Within BPD, this may help to explain why participants in this study described negative and concrete but also positive and understanding experiences with others at different times (Fonagy & Luyten, 2010, p. 261).

### Hurt and healing

Findings highlighted the complex and intertwined nature of traumatic experience, personality organization, and self/identity. Often participants described an inherent sense of helplessness to change the abusive relationships they found themselves in as children and also a continuation of abuse from childhood to adulthood. Participants also demonstrated strands of empowerment within their stories by describing helpful encounters with others, autonomous decisions and healing experiences.

Although a contentious theory, results from the participants in the study demonstrate many parallels to Herman's (1992) conception of complex trauma and recovery in describing repeated abuse in childhood as having a direct impact on personality development in forming and deforming a child's sense of self. The conceptualization of the abusive familial environment has many similarities to the environments described by participants including terrible circumstances, helplessness, unpredictability, and the need for constant alertness (p. 96–114). Narratives also described the seeds of recovery theory in overcoming disconnection and disempowerment through empowerment, personal responsibility and new connections (p. 133–154).

### Implications and future suggestions

#### Connecting to myself

Findings suggest that identity is an integral aspect of BPD difficulties. Psychiatric diagnosis and traditional psychological theories and interventions have been proposed to oversimplify the concept of identity leading to insufficient understandings of challenges and subsequently ineffective interventions (Miller, 1994). Results of this study give support to therapies that focus on healthy aspects of the self (Giesen-Bloo et al., 2006) and improve narrative coherence in BPD (Clarkin, Levy, Lenzenweger, & Kernberg, 2007). An emphasis on narrative coherence in other PDs may be beneficial in understanding narrative identity in light of increasing calls to assess PDs as a spectrum of difficulties (Shedler & Westen, 2004).

#### Distance between us

Studies often report that aspects of the therapeutic relationship are the biggest predictor of positive therapeutic outcomes across psychotherapies (Lambert & Barley, 2001; Wampold, Minami, Baskin, & Callen, 2002). For therapists, individuals with BPD can often be termed as “difficult to treat” due to the sudden changes in relationship patterns and a sense of helplessness and rejection that can potentially be evoked in the therapist (Ryle, 2007). Results of this study suggest that individuals with BPD have differing abilities to connect with others ranging from disconnection to intimacy. Therapeutic understanding that takes into account the deficiencies within diagnostic criteria and acknowledges the diverse nature of self and identity of an individual may improve the therapeutic relationship.

Results suggested that narratives associated with change were mainly absent although they did occur. Despite participants demonstrating connection and insight into their relationship patterns on some occasions, of note was the lack of narratives associated with change following insight. This is an anomaly also found within the narrative study by Adler et al. (2012) when they described the small sense of agency and a lack of change narrative. This finding is contrary to the consensus within the psychotherapy literature that realization has the potential to prompt therapeutic change (Jinks, 1999). Theoretical understandings of what insight and self-awareness are and how different theoretical models attempt to increase them and produce change differ widely (Bateman et al., 2007; Levitt et al., 2004). A lack of perceived control was highlighted by results, for example, Hurt and healing. These are perceptions which are often lacking in BPD populations. Dialectical behaviour therapy is an example of a therapy which has gained acclaim as a treatment of BPD and does not directly relate meaning and insight to therapeutic change. It emphasized radical acceptance and behavioural change over deriving meaning and insight into relationships, behaviours, and cognitions (Palmer, 2002).

Research in the area of dependency, change and insight within BPD populations is warranted. Focusing on dependency styles in association with BPD may give therapists an idea of how likely gaining new insight may impact upon tangible change, for example, reduction of life threatening behaviour, increases in quality of life, and overall improvements in functioning.

#### Hurt and healing

Similarities and parallels exist between the narratives of participants and the conceptualization of Herman's (1992) complex trauma. Participants’ narratives highlighted a non-linear progression between gaining stability in their lives, looking back on their past experiences, and mending relationships and forging new relationships. This cyclical pattern can be viewed within Herman's complex trauma model and the phase-orientated intervention that has been further developed since this time. It may be helpful for therapists to refer to the stages of recovery model or consider a phase-orientated approach (Courtois, Ford, & Cloitre, 2009) when working with the BPD population with an abuse history.

### Limitations

This study attempted to provide exploratory research into an inherently complex area. The study of personal narratives focuses on the personal subjective meaning that an individual derives from their life. Whenever self-report data is collected there is the potential for a difference between what is reported and the reality it allegedly describes (Adler et al., 2012). Also, the present study focused solely on five individual females. While we view this as a strength allowing a rich and in-depth investigation of participant identities, it also restricts the potential generalizability of our findings, for example, males with a BPD symptomology.

The design of the present study does not allow for an assessment of the relationship direction between identity and BPD status. It is possible that BPD symptoms lead to narrative identity disruptions, that narrative identity disruptions result in the development of BPD symptoms, or that other variables are responsible. It is possible that the clusters of themes identified in this study are not distinctive to the BPD population but may represent general themes of identity relating to comorbid presentations, non-clinical or other clinical samples. This highlights a gap in the research base and future research may focus on the most general area of identity or replicate this study and also control for these factors.

## Conclusions

Findings provided support for the theoretical conceptualization of self/identity that are multiple and flexible in comparison to the idea of a unitary self/identity. Results also suggested that participants were able to establish differing connections to others ranging from disconnection to intimacy and care. Participants reported that their identities were impacted upon by historical and current family/relationship dysfunction, but life stories also illustrated the positive effect of healing relationship experiences. Findings supported theories and interventions that consider a multiple and relational self and the empowerment of healthy aspects of the self in BPD recovery. Studies that assess the association between insight and change may add further knowledge to this complex population.
